# An X-ray chamber for *in situ* structural studies of solvent-mediated nanoparticle self-assembly

**DOI:** 10.1107/S0909049513001143

**Published:** 2013-02-06

**Authors:** Davide C. E. Calzolari, Diego Pontoni, Jean Daillant, Harald Reichert

**Affiliations:** a European Synchrotron Radiation Facility, Beamline ID15, 38043 Grenoble, France; b CEA, IRAMIS, LIONS, Batiment 125, CEA Saclay, F-91191 Gif-sur-Yvette Cedex, France; c Max-Planck-Institut für Metallforschung, Heisenbergstrasse 3, D-70569 Stuttgart, Germany

**Keywords:** X-rays, self-assembly, nanoparticles, solvents, wetting, *in situ*

## Abstract

Spontaneous ordering of nanoparticles (NPs) occurring as a consequence of solvent evaporation can yield highly ordered and extended NP superlattices bearing both fundamental scientific interest and potential for technological application. A versatile experimental chamber has been developed allowing (i) controlled *in situ* deposition of NP solutions on solid substrates, (ii) rate-controlled evaporation of the bulk solvent, and (iii) adsorption/desorption of nano-thick solvent films onto preformed NP assemblies. Within this hermetically sealed chamber all the stages of self-assembly, including macroscopic solution evaporation, NP thin-film formation and its subsequent structural transformation induced by nano-thick solvent films, can be characterized *in situ* by X-ray scattering techniques. Here, technical design and calibration details are provided, as well as three experimental examples highlighting the chamber’s performances and potential. Examples include the controlled adsorption of thin toluene films on flat silicon wafers, the observation of transient accumulation of gold NPs near the toluene–vapour interface, and preliminary data on the structural effects of fast macroscopic solvent evaporation followed by nanoscale solvent adsorption/desorption from a vapour phase. By combining bulk evaporation rate control, fine tuning of the thickness of adsorbed solvent films and *in situ* X-ray characterization capabilities, this cell enables explorations of both near-to-equilibrium and far-from-equilibrium routes to NP self-assembly.

## Abstract 2

Technical details and experimental examples are given for a novel sample environment allowing X-ray investigations of self-assembly phenomena induced by controlled bulk solvent evaporation and nanoscale solvent adsorption/desorption.

## 1. Introduction   

New materials based on nanoparticles (NPs) are expected to play a key role in the development of future nanotechnology (Urban *et al.*, 2007 ▶; Jin *et al.*, 2008 ▶; Tseng *et al.*, 2005 ▶). One of the main challenges in this respect is the development of facile and reproducible methods for directing the organization of NPs into macroscopically extended (∼millimeters) ordered arrays and superlattices. The exploitation of spontaneous ordering phenomena, often referred to as *self-assembly* (Whitesides & Grzybowski, 2002 ▶; Kinge *et al.*, 2008 ▶; Grzelczak *et al.*, 2010 ▶), represents a promising route towards the accomplishment of this goal.

Among various possible approaches, increasing attention has been devoted during the last 15 years to NP self-assembly processes occurring as a consequence of solvent evaporation (Murray *et al.*, 1995 ▶; Fendler, 1996 ▶; Shevchenko *et al.*, 2006 ▶; Nie *et al.*, 2010 ▶). During the evaporation of the solvent in which the NPs are dissolved, a complex interplay between various types of interparticle interactions and drying kinetics effects can lead to the formation of a plethora of self-assembled structures characterized by a rich variety of nanoscale architectures (Pauliac-Vaujour & Moriarty, 2007 ▶; Martin *et al.*, 2007 ▶; Lin *et al.*, 2001 ▶; Bigioni *et al.*, 2006 ▶).

Typical investigations often consisted of depositing droplets of NP dispersions onto solid substrates, letting the solvent evaporate in open-air conditions and characterizing the resulting dry NP film with *ex situ* microscopy techniques. In several cases this simple approach led to the discovery of essential features of NP self-assembly (Ohara *et al.*, 1995 ▶; Kiely *et al.*, 1998 ▶). Nowadays, *in situ* studies under precisely controlled solvent conditions are becoming increasingly important in order to achieve a detailed understanding of various NP self-assembly mechanisms. In this respect, synchrotron X-ray characterization techniques are very useful, as demonstrated for example by recent investigations of colloidal phase transitions and NP self-assembly processes both in bulk and at interfaces (Pontoni *et al.*, 2003 ▶, 2009 ▶; Narayanan *et al.*, 2004 ▶; Daillant, 2009 ▶; Roth *et al.*, 2010 ▶; Giner-Casares *et al.*, 2012 ▶).

Of particular relevance for the work presented here is the annealing of preformed NP assemblies *via* controlled solvent adsorption from a vapour phase (Alvine *et al.*, 2006 ▶). Unlike uncontrolled drying of NP dispersions, this approach allows investigations of NP structures that are still wet by thin solvent films in near-equilibrium stable conditions. For example, nanoscale structural transformations of gold NP monolayers induced by thin adsorbed solvent films were recently observed by *in situ* X-ray scattering and *ex situ* microscopy techniques (Pontoni *et al.*, 2009 ▶).

In this context we developed a versatile experimental chamber allowing *in situ* studies of solvent-mediated NP self-assembly. The chamber described here represents the most recent evolution of a sample environment concept that was initially developed for the investigation of the wetting properties of pure liquids on model surfaces (Tidswell *et al.*, 1991*a* ▶,*b* ▶; Gang *et al.*, 2002 ▶, 2005 ▶; Fukuto *et al.*, 2006 ▶; Hofmann *et al.*, 2010 ▶), and subsequently applied to the study of solvent-induced annealing of *ex situ* assembled NP structures (Alvine *et al.*, 2006 ▶; Pontoni *et al.*, 2009 ▶). The most important development in the design presented here is the injection system allowing the direct deposition of bulk NP solutions onto solid substrates mounted inside the hermetically sealed cell. The ability to inject NP solutions without breaking the chamber’s hermetic sealing enables the investigation of both the formation of evaporation-induced NP assemblies *and* their structural annealing by controlled solvent adsorption and desorption. All the intermediate states of the NP system, from the initial dilute bulk suspension to the final dense and dry NP film, become therefore accessible to *in situ* X-ray characterization using techniques such as X-ray reflectivity (XR) and grazing-incidence small-angle X-ray scattering (GISAXS).

In the next section we recall the chamber’s working principle and describe its characteristics and design details. In the subsequent sections the wetting cell performance is demonstrated by three experimental examples: (i) the determination of the effective Hamaker constant for the interaction between toluene and silicon oxide, which was performed as a validation test before applying the chamber to the study of NP-based systems, (ii) the observation of an unexpected transient NP accumulation near the surface of freshly injected dilute suspensions of thiol-stabilized Au NPs in toluene, and (iii) preliminary results concerning the NP self-assembly processes occurring after fast evaporation of the bulk solvent followed by slow nanoscale solvent adsorption and desorption.

**Figure 1:**
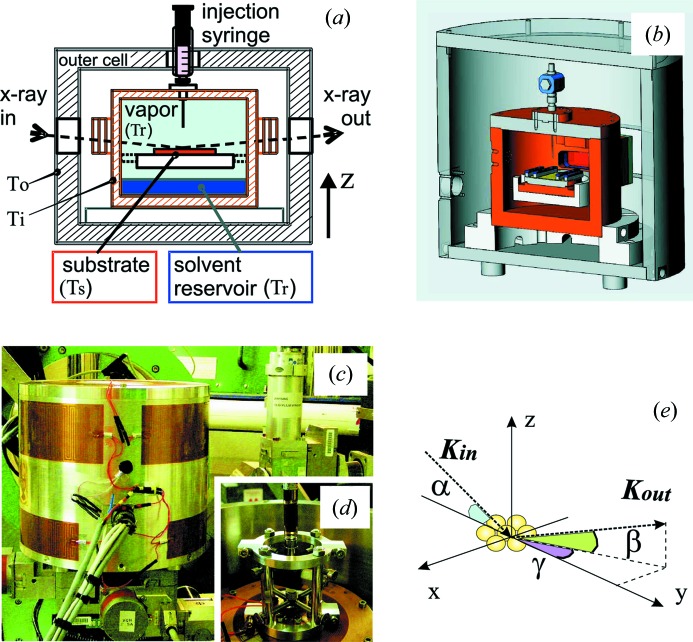
(*a*) Schematic cross section of the wetting cell system: tuning 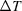 = 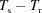 allows control of both bulk solvent evaporation and nanoscale solvent adsorption and desorption from the vapour phase onto the solid substrate. (*b*) True side section [rotated by ∼90° with respect to (*a*)] obtained from the drawing axonometric projection. (*c*) Photograph of the cell assembled and mounted on the sample stage of a synchrotron beamline. (*d*) Detail of the hermetically sealed NP-solution injection system. (*e*) X-ray scattering geometry.

All the experiments were carried out at the European Synchrotron Radiation Facility in Grenoble, France. The toluene-SiO_2_ Hamaker constant was determined *via* *in situ* XR measurements at the beamline ID15C (de Jong, 2010 ▶), using an X-ray beam delivering ∼5 × 10^8^ photons s^−1^ in a focal spot of 5 µm × 500 µm (V × H) at an X-ray energy of 38.7 keV. The NP systems were investigated at the ID15A beamline (Reichert *et al.*, 2003 ▶; Honkimäki *et al.*, 2006 ▶) using an X-ray beam delivering ∼5 × 10^11^ photons s^−1^ in a focal spot of 5 µm × 20 µm at an X-ray energy of 69.8 keV. The reflected beam intensity was measured using a scintillation detector system (Cyberstar). In the following the X-ray data sets are presented as a function of the wavevector transfer *q*, whose Cartesian components are *q*_*x*_ = 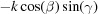, *q*_*y*_ = 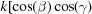 − 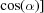 and 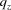 = 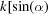 + 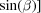, where the angles 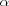, 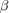 and 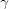 are defined in Fig. 1(*e*) ▶, *k* = 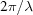 and 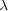 is the X-ray wavelength.

## 2. Working principle and chamber implementation   

The sample environment developed for our investigations is composed of an external stainless steel vessel and an internal copper chamber (Fig. 1 ▶). The external shell isolates the inner cell from ambient temperature variations and is used to pre-heat the whole system. Its temperature is typically set 2–5 K lower than the target inner-cell temperature. The inner cell is the core of the apparatus and contains the substrate onto which the NPs are induced to self-assemble. The inner cell consists of a hermetically sealed copper chamber designed to enable the precise control of both *macroscopic* solvent evaporation and *microscopic* solvent adsorption/desorption onto bare or NP-loaded substrates.

NP dispersions can be injected directly onto a horizontally lying substrate mounted on a sample holder suspended inside the inner chamber [Figs. 1(*a*) and 1(*b*) ▶]. A system comprising a welded needle, a precision valve and a syringe-holder frame (Fig. 1*d* ▶) allows injection of the NP solution without breaking the inner cell’s hermetic sealing. Increasing rates for the bulk solvent evaporation can be selected by increasing the positive temperature difference between sample holder and inner cell, and by adjusting the power delivered by the sample heater.

The absorption of nanoscale solvent wetting films onto the substrate (Tidswell *et al.*, 1991*a* ▶; Heilmann *et al.*, 2001 ▶; Gang *et al.*, 2002 ▶; Pontoni *et al.*, 2009 ▶; Hofmann *et al.*, 2010 ▶) is achieved by controlling with ∼1 mK precision the temperature difference 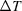 = 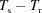 between the substrate to be wet (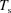) and a liquid solvent reservoir [dark blue, Fig. 1(*a*) ▶] whose temperature 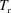 is determined by the inner cell temperature 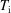. The sample holder is suspended above the bottom of the cell and is fixed to its internal wall (Fig. 1*a* ▶). The liquid reservoir is injected at the cell bottom and is not in contact with any part of the sample holder (Fig. 1*a* ▶). Therefore, transfer of liquid from the solvent reservoir to the substrate is only possible through the vapour phase [sky blue, Fig. 1(*a*) ▶], which is in equilibrium with the reservoir at temperature 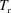.

The precise control of 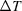 allows regulation of the chemical potential offset from the bulk liquid–vapour coexistence 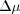 ≃ 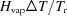, where 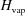 indicates the heat of vaporization of the solvent (Tidswell *et al.*, 1991*a* ▶; Gang *et al.*, 2002 ▶). Neglecting tiny gravitational field contributions, the equilibrium thickness 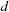 of the film adsorbed onto the substrate depends on the balance between: (i) the chemical potential offset 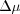 that determines an energetic cost for the film formation, and (ii) the van der Waals attraction between the solvent molecules and the substrate, favouring film growth (Gang *et al.*, 2002 ▶; Fukuto *et al.*, 2006 ▶). We can thus express the total free energy excess per unit area as 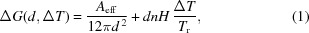
where 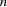 is the solvent molecular number density and 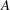
_eff_ is the effective Hamaker constant for the specific solvent–substrate interaction. The thermodynamic equilibrium thickness 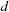 of the film wetting the substrate is determined by the condition 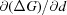 = 0, yielding
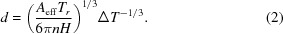
For typical values of 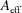 of the order of 10^−20^ J, solvent films with stable equilibrium thickness ranging from a few Å to about 10 nm can be formed as 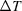 varies from ∼10 K to ∼1 mK, respectively.

In order to obtain a solvent film of stable thickness over a long period of time, three important conditions have to be fulfilled: (i) the system has to be hermetically sealed during all the operations, in order to maintain a stable vapour pressure; (ii) each element needs to be thermally very stable, in principle within 1 mK precision, over a long time [equation (2)]; (iii) the system has to be thoroughly cleaned to avoid slow and continuous dissolution of impurities in the solvent reservoir.

Various approaches are used to ensure the hermetic sealing of the inner cell. The cell lid is firmly clamped against the cell body using eight stainless steel screws and a gasket made of indium wire, which is freshly replaced every time the system is closed to start a new experiment. The connections between the injection needle, the valve and the syringe (Fig. 1*d* ▶) are also leak-tight. The X-ray beam reaches the sample after traversing the outer-cell Kapton windows and then the inner-cell beryllium (Be) windows (Fig. 1*a* ▶). The latter are conceived to ensure a good thermal and pressure insulation. They comprise two 200 µm-thick rectangular (18 mm × 53 mm) Be foils clamped between two copper holders and sealed from one side by means of a Teflon (PTFE) frame and from the other side by an indium sheet washer. As common in X-ray applications, Be was chosen for its low atomic number and very low absorption for X-rays (absorption coefficient μ = 0.267 cm^−1^ at 70 keV, μ = 1.884 cm^−1^ at 8 keV), together with a high Young’s modulus 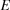 = 289 GPa. The 200 µm thickness ensures mechanical stability up to a pressure of ∼30 kPa (Young, 1989 ▶), which represents the maximum vapour pressure allowed inside the inner cell. In the case of toluene, the solvent used in our experiments, this maximum vapour pressure corresponds to a maximum 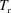 ≃ 343 K (Lide, 2004 ▶). Our typical inner-cell working temperature is between 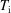 = 301 K and 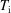 = 308 K.

Efficient and uniform heating of the inner and outer cells is obtained by means of a series of Kapton flexible resistive heaters (MINCO) glued onto their external surfaces. One additional adhesive-free round-shaped heater is sandwiched between the copper sample holder and the underlying support at the interior of the inner cell. This heater allows the temperature of the sample substrate (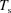) to be increased with respect to that of the vapour phase (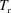). The inner- and outer-cell temperatures (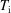 and 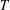 ), as well as 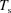 and 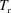, are measured by high-precision epoxy thermistors (YSI) having a nominal resistance of *R* = 30 KΩ at 298 K. The sample and vapour thermistors are connected to the exterior *via* two electrical feedthroughs of ultra-high-vacuum quality. The sample thermistor is inserted inside a guide hole pierced through the copper sample holder such that the thermistor’s head is positioned right beneath the centre of the sample substrate.

**Figure 2:**
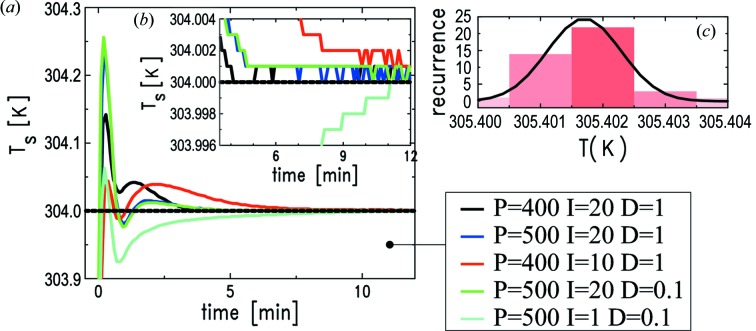
(*a*) Refinement of the PID settings for the sample heater control loop. The configuration ensuring faster attainment of the setpoint (304.0 K) and minimal temperature overshoot corresponds to the black curve. (*b*) Same data as in (*a*) over the 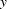-axis range (setpoint ± 4) mK. (*c*) Gaussian distribution of 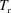 values measured over a period of 2 h after reaching thermal stabilization: the standard deviation is 0.6 mK.

The thermal control is achieved by means of three feedback loops controlled by two Lakeshore temperature controllers (models 336 and 340). An external power supply (Kepco, 75 V maximum voltage) can also be interfaced with the Lakeshore controllers, in order to apply, if needed, a higher heating power to the sample, thus reaching higher solvent evaporation rates. We have optimized the proportional, integral and differential (PID) parameters of the three loops used to control 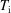, 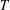  and 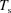 (Fig. 2*a* ▶). The PID optimization is important in order to stabilize the temperatures to the setpoint within the shortest possible time and with the smallest transient temperature overshoot. The Gaussian fit of the temperature histogram in Fig. 2(*c*) ▶ demonstrates that the control precision achieved for 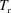 is better than 1 mK (FWHM = 0.0006 K). The same degree of stability is reached for 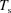, therefore the apparatus enables control of 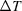 to within 1.2 mK.

**Figure 3:**
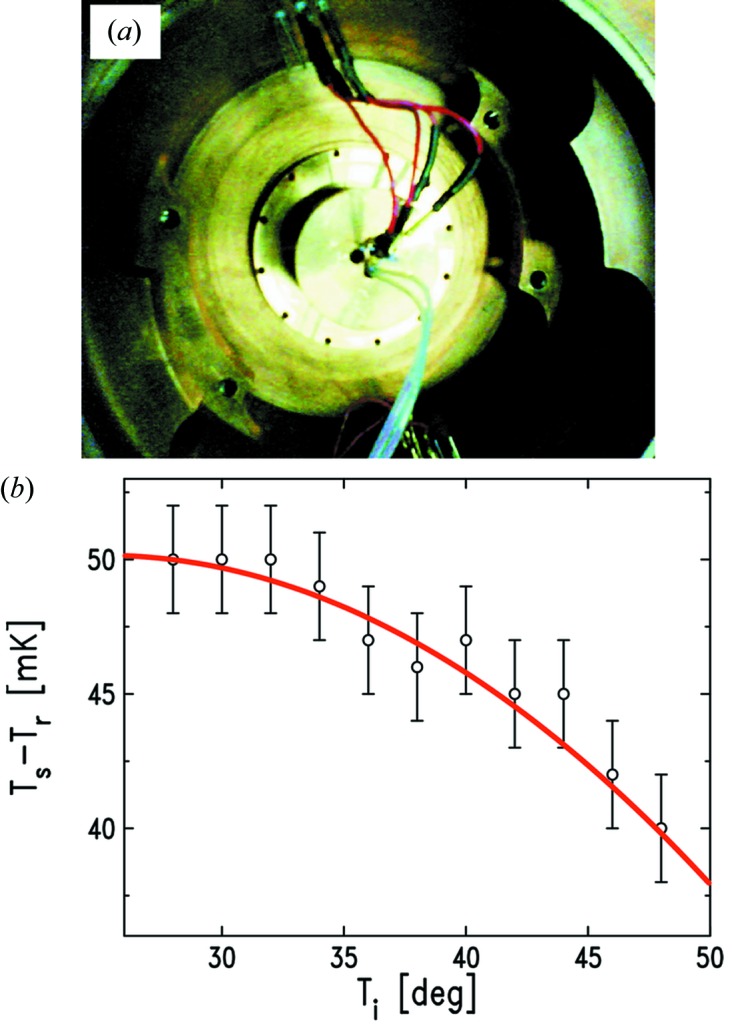
(*a*) Vapour and sample thermistors in place inside the copper block for the calibration. (*b*) Result of the measurement: the offset is of 50 mK in our usual working region (301 K < *T* < 307 K) and it decreases with a quadratic dependence on 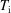.

The accurate determination of 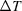 = 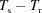 requires also the cross-calibration of the substrate and vapour thermistors’ response. In fact, despite using thermistors of exactly the same type, any two of them always exhibit slight discrepancies (a few tens of mK) in the measurement of the same temperature. We therefore measure this response offset by inserting the two thermistors inside two adjacent holes drilled in a bulky copper block placed at the centre of the sealed wetting chamber (Fig. 3*a* ▶). Various 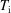 values are set within our region of interest (298–323 K) and the complete thermal stabilization of the chamber is awaited before storing the values yielded by the two thermistors. An example of a resulting calibration curve is reported in Fig. 3(*b*) ▶, where it is evident that in this particular case the thermistor response offset was ∼50 mK at room temperature, and that it tended to decrease quadratically with increasing 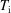. This thermistor cross-calibration procedure is repeated whenever either the sample or vapour thermistor is replaced.

Inside the inner cell the substrate is positioned onto a sample holder, which is suspended in order to avoid any contact with the solvent reservoir. This holder has also the important role of thermally insulating the substrate from the body of the inner copper cell, thus allowing to establish stable non-zero 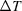 values. Three different sample holders have been fabricated using materials (PTFE, stainless steel and copper) characterized by different thermal conductivity (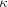) values. This allows one to choose among different degrees of thermal insulation according to the required experimental conditions. Table 1 ▶ lists the relevant thermophysical properties of the three chosen materials, whose thermal insulation power (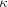) ranges over about three orders of magnitude.

**Figure 4:**
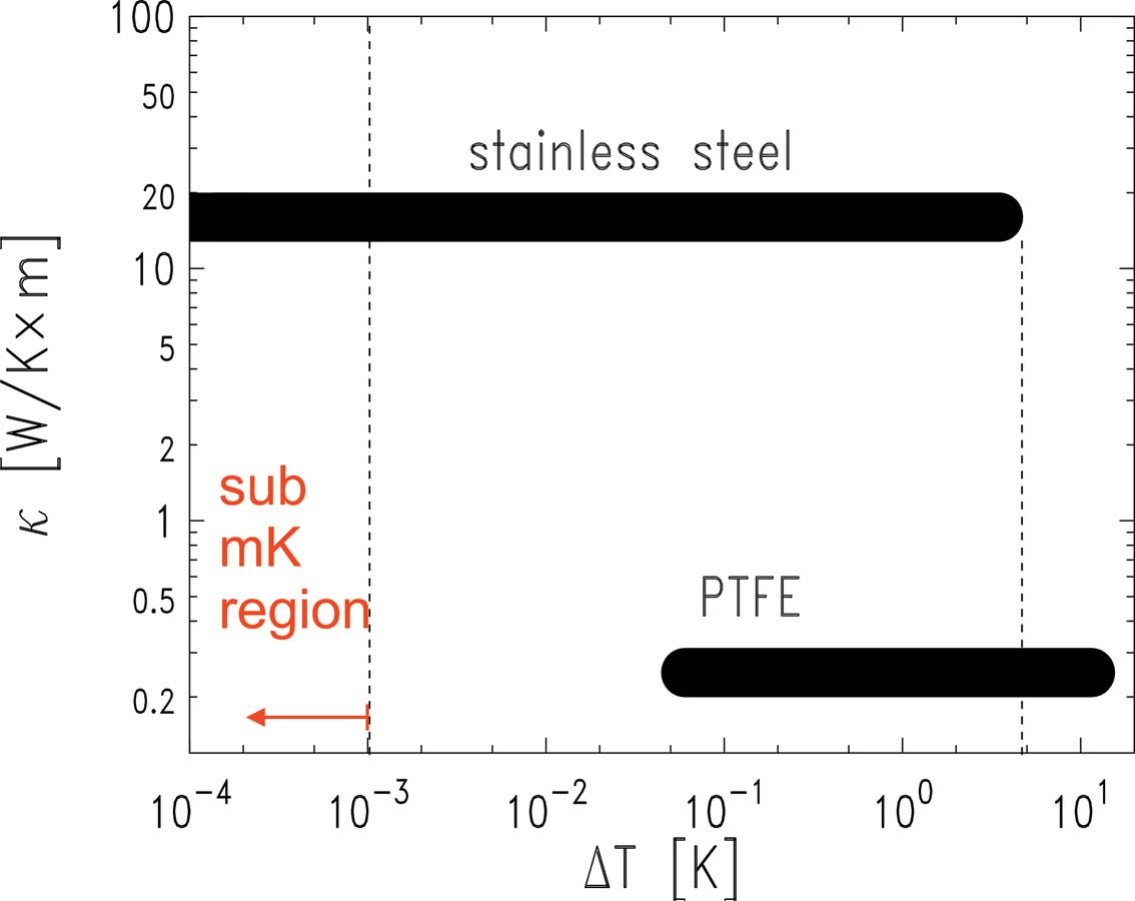
Ranges of stable 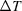 obtainable by using the PTFE and the stainless steel sample holders.

Fig. 4 ▶ shows the sub-ranges of 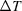 covered with the PTFE holder and with the stainless steel holder. The copper sample holder has the same thermal conductivity as the inner cell body, therefore its thermal insulation ability is minimal and it allows only very small 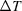 values to be reached, which are comprised within the range covered by the stainless steel holder. For this reason the copper holder was not used in the experiments presented here. Owing to its low 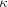 value, the PTFE holder allows 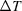 ≃ 12 K to be reached, which is expected to correspond to a few Å of adsorbed toluene film thickness on silicon oxide. However, this low thermal conductivity makes it practically impossible to achieve stable 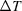 values smaller than ∼60 mK. Conversely, with the stainless steel sample holder it is not possible to set stable 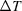 values greater than ∼3.5 K, but the condition 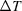 ≃ 0 mK can be easily reached.

In order to exclude the presence of any contaminant, all the inner-cell parts undergo a thorough cleaning procedure involving 2-propanol, acetone and chloroform before each experiment. The sample substrate is sonicated for 15 min in each of these three solvents, then immersed for 10 min in Piranha solution, and finally thoroughly rinsed under flowing ultra-pure deionized water. The sample is then dried under a stream of ultra-pure argon, and finally UV-irradiated in an O_3_ atmosphere for at least 15 min to let the ozone eliminate any residual organic material that may have adsorbed onto the sample surface. Finally, the substrate insertion and cell lid sealing are performed inside a glove box under ultra-pure N_2_ atmosphere.

## 3. Controlled nanoscale solvent adsorption and desorption on flat solid surfaces   

As a first characterization of the wetting cell performance, we studied pure solvent wetting onto a bare and flat solid surface. Using a toluene reservoir at the bottom of the inner cell (Fig. 1 ▶) and selecting different stable 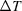 values, solvent films of different thickness were adsorbed onto a low-roughness silicon (Si) wafer covered by a native silicon oxide (SiO_2_) layer. Chromatography-purity toluene (Sigma-Aldrich) was used as received.

In order to evaluate the thickness 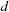 of the solvent layer adsorbed, we have fitted the measured XR curves using a simple box model approach, where the average intrinsic electron density profile of the system 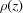 is obtained by a sum of 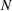 boxes of thickness 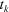 and electron density 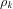, each one representing a different layer (Fukuto *et al.*, 2004 ▶),
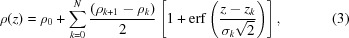
where 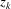 and 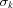 represent, respectively, the position and the roughness of the layer-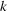
–layer-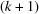 interface, and the interfacial roughness is described by an error function. In particular, 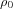 is the electron density of the bulk substrate, 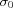 and 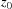 = 0 are, respectively, the roughness and *z*-coordinate of its surface, and 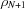 is the electron density of the bulk phase present above the sample (superphase). To a good approximation 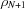 = 0 when the superphase is a vapour. The thickness of layer 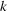 is 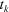 = 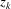 − 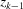 (with 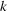 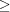 1 and 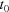 = 0).

**Figure 5:**
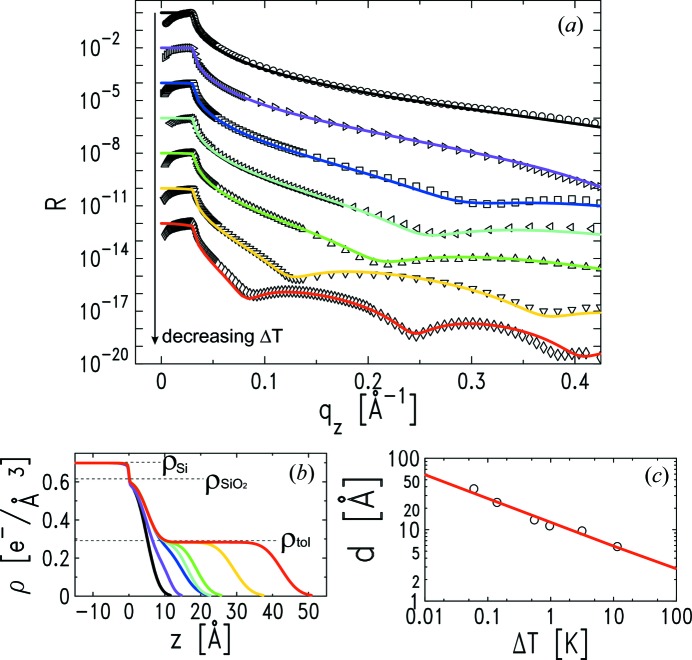
(*a*) X-ray reflectivity profiles corresponding to the dry silicon substrate (circles) and to the toluene film adsorbed at 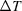: 11.452 K (right triangles, 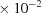), 3.150 K (squares, 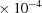), 963 mK (left triangles, 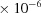), 551 mK (up-triangles, 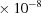), 142 mK (down-triangles, 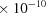) and 60 mK (diamonds, 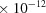). The solid lines are two-box model fits yielding the electron density profiles presented in (*b*). (*c*) Fitted thickness of the wetting film as a function of 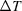 in log–log representation. The power-law fit (red line) to the data is consistent with the expected 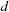 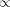 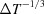 dependence.

The first XR curve (circles) plotted in Fig. 5(*a*) ▶ is measured on the dry Si substrate surface, before injecting the toluene reservoir. This curve can be fitted using a single-box model [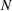 = 1 in equation (3)], where the substrate is silicon (electron density 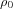 = 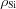 = 0.699 e^−^ Å^−3^) and the single box represents a thin native silicon oxide (SiO_2_) layer. The formation of this oxide layer is expected since the sample is not in a vacuum (Tidswell *et al.*, 1990 ▶). The Si/SiO_2_ layer interface roughness 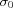 was fixed to 0 Å (Tidswell *et al.*, 1990 ▶), which amounts to assuming that the interface is perfect. A good description of the experimental data is obtained by considering a SiO_2_ layer of thickness 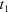 = 5.1 Å, roughness 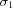 = 2.7 Å and electron density 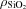 = 0.609 e^−^ Å^−3^ [black line fit of Fig. 5(*a*) ▶].

The other XR curves of Fig. 5(*a*) ▶ are measured after injecting the toluene reservoir in the cell, at decreasing 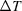: 11.452 K, 3.150 K, 963 mK, 551 mK, 142 mK and 60 mK. Since we performed this test with the PTFE sample support mounted, 60 mK is the smallest 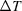 reached. For each stable 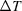, XR measurements were repeated at different lateral locations on the sample and at subsequent times, in order to check the spatial homogeneity and the temporal stability of the adsorbed solvent film. These two requirements are both fulfilled, confirming 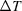 stability and the efficiency of the hermetic sealing and cleaning procedure.

The XR measurements of the toluene-wet substrate are fitted using a two-box model [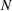 = 2 in equation (3)], where the first and second boxes describe the native SiO_2_ layer and the adsorbed toluene layer, respectively. In order to fit the toluene film thickness *d* = 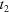, the Si and toluene electron densities were fixed to their respective nominal values 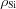 = 0.699 e^−^ Å^−3^ and 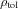 = 0.283 e^−^ Å^−3^. 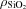 and 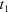 were fixed to the respective values inferred from the dry-silicon fit. The SiO_2_/toluene interface roughness was left free to vary in a small range around the previously inferred 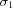 = 2.7 Å. The second-box roughness 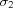, corresponding to the toluene film/vapour roughness, was left free to vary in a small range around the thermally induced capillary wave roughness 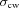. 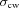 of bulk toluene for our experimental 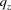-resolution of 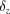 = 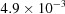
 Å^−1^ [slit aperture 0.25 mm × 1.6 mm (V × H)] and for 307 K < *T* < 320 K (the range covered by 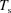 in the experiment) ranges between 3.9 Å and 4.2 Å (Braslau *et al.*, 1988 ▶). This is an approximation, since the system under examination is not a bulk liquid but a thin solvent film, and the van der Waals liquid–substrate interaction should be taken into account (Tidswell *et al.*, 1991*b* ▶).

The fitted parameters for the second box, describing the adsorbed toluene film, are listed in Table 2 ▶. The electron density profiles are presented in Fig. 5(*b*) ▶, and the corresponding reflectivity fits in Fig. 5(*a*) ▶. The fitted toluene film thickness is plotted as a function of 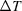 in log–log representation in Fig. 5(*c*) ▶. The experimental points are fitted with a power law (red line) having exponent −0.34 ± 0.04, which agrees with the expected value of −1/3 [equation (2)].

From the power-law fit we can estimate (Tidswell *et al.*, 1991*b* ▶) also the effective Hamaker constant for the interaction of toluene with oxidized silicon (SiO_2_). The Hamaker constant 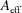 appears in the proportionality constant that links 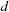 to 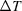 [equation (2)]. Considering the heat of vaporization of toluene, 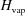 = 38.6 kJ mol^−1^ (Svoboda & Mayer, 1985 ▶), and the experimental solvent vapour temperature of 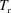 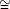 307 K, we obtain 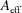 = (4.5 ± 1.3) × 10^−20^ J for the toluene–silicon-oxide interaction. We could not find other experimental values for this constant in the literature; however, in the Lifshitz theory approximation (Israelachvili, 1985 ▶) the expected Hamaker constant for the toluene–silicon-oxide interaction is calculated to be 6.4 × 10^−20^ J, which is slightly above the upper limit of our experimental result.

Previous XR investigations of nanoscale liquid films suggested the existence of liquid density anomalies at the substrate–liquid interface (Doerr *et al.*, 2000 ▶). With the maximum 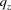 values reached in the first set of test measurements presented here we could not resolve the density profile of the liquid with the required resolution to confirm the existence of those density anomalies. Using the average density of toluene as a free parameter, we found, however, indications for a slight density deficit in the thin liquid toluene films adsorbed on oxidized silicon.

## 4. XR measurements of stable NP-solution–vapour interfaces   

By means of the injection system described above (Fig. 1 ▶), we deposited 2 ml of NP dispersion onto a 2-inch sapphire wafer mounted within the wetting chamber. We used Au NPs, sterically stabilized by a coating shell of decane-thiol molecules, and dispersed in toluene. The average NP core size was 6.5 nm, with size polydispersity ∼10%, determined by small-angle X-ray scattering measurements (Pontoni *et al.*, 2002 ▶). The injection was performed at a stable 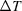 = 60 mK. The concentration of the solution, 0.34 µ*M*, was adjusted such that each millilitre of dispersion contained the number of NPs needed to homogeneously cover the wafer with a monolayer of hexagonally close-packed NPs. The amount of solution injected, 2 ml, ensured spreading over the complete surface of the wafer and pinning of the liquid at the wafer edges.

The macroscopic thickness of the deposited solution was checked right after injection by simply scanning the cell vertically along the 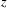 direction (Fig. 1*a* ▶) across the X-ray beam, and measuring its attenuation. The initial thickness of the solution, measured at the centre of the sample, was ∼1100 µm. Approximating the volume occupied by the NP solution as a 2-inch-diameter cylinder (the diameter of the sapphire wafer), the expected thickness for the 2 ml of NP solution injected would be ∼990 µm. Therefore the entire volume injected did actually remain on the substrate, pinned at the edges of the sapphire wafer, as observed in preliminary *ex situ* injection tests. The fact that the measured liquid thickness is slightly larger than expected is mainly due to the approximation adopted by describing the solution as a perfect cylinder. The thickness of the bulk NP solution was measured repeatedly at regular time intervals. This allowed detecting a decrease of the liquid–vapour interface height of less than 2 µm per hour, therefore confirming the very low toluene evaporation rate expected at 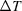 = 60 mK.

**Figure 6:**
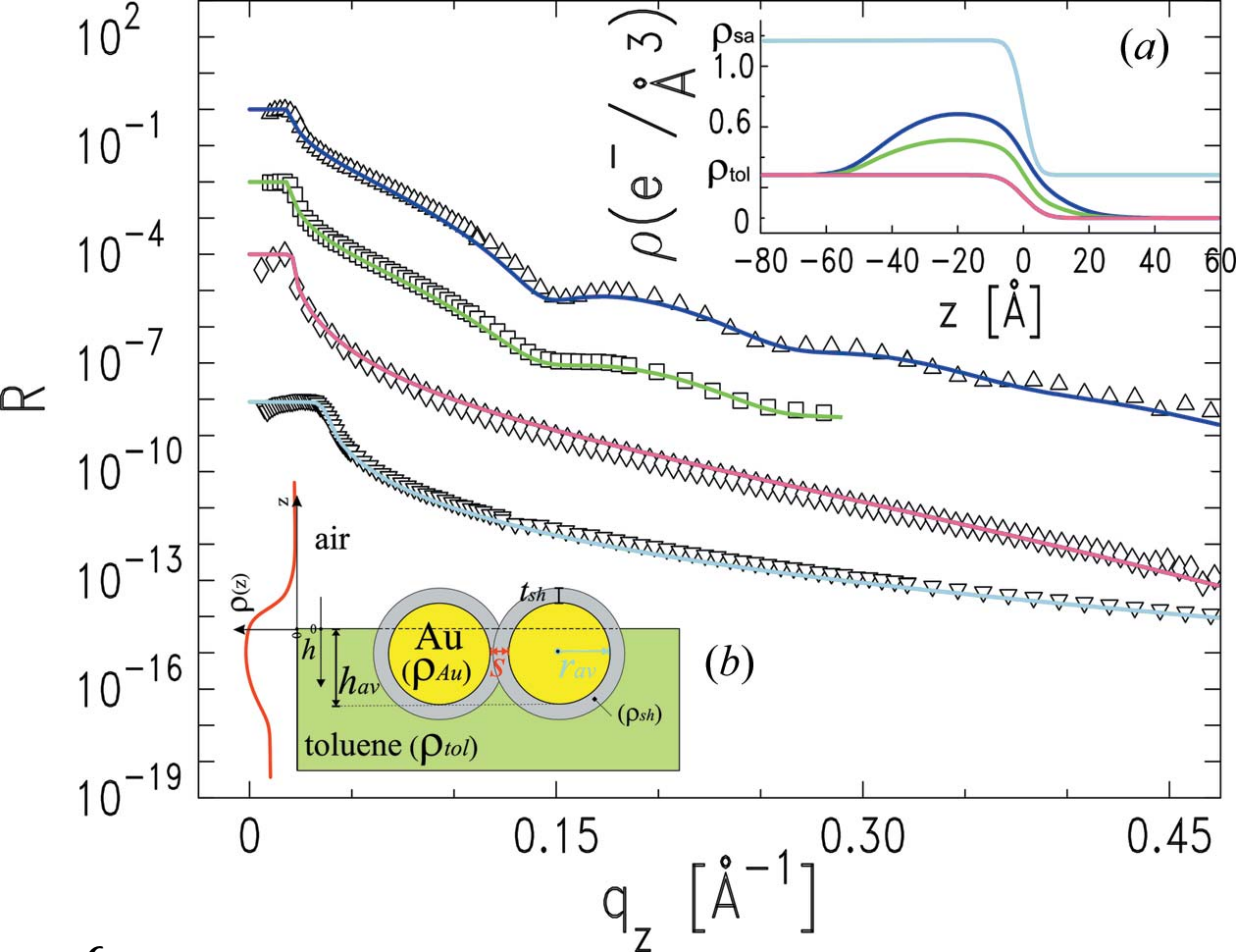
Experimental XR curves from a 2 ml bulk NP solution injected onto a 2-inch-diameter sapphire substrate (bulk electron density 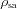 = 1.175 e^−^ Å^−3^) and kept at constant 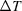 = 60 mK for ∼20 h. The solution contains the NPs necessary to assemble a close-packed NP bilayer covering the entire sapphire wafer after complete solvent evaporation. Up-triangles: NP solution surface 5 h after injection. The oscillatory XR character indicates surface NP accumulation. Squares: (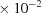) same NP solution 8 h after injection. The smaller oscillation amplitude suggests partial NP dissolution into the bulk sub-phase. Diamonds: (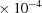) same NP solution 18 h after injection. The absence of XR oscillations indicates the complete disappearance of the transient surface NP layer. Down-triangles: (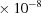) example of XR measurement at the buried substrate–NP-solution interface. The monotonic XR character indicates the absence of NP accumulation at the buried solid–liquid interface. The solid lines are physical model fits obtained by applying the Parratt recursive method to the electron density profiles reported in inset (*a*). Inset (*b*): schematic representation of two core-shell NPs at the toluene–vapour interface, with the parameters involved in the physically motivated model described in §4.

We first investigated by XR the surface structure of the NP solution deposited according to the above-described procedure. Fig. 6 ▶ summarizes the result of this first investigation. The top data set (up-triangles) represents the first XR profile measured at the NP-solution–vapour interface, about 5 h after injection. The oscillatory character of this XR profile is the signature of a near-surface nano-thick high-electron-density layer. A simple box model fit (not shown) indicates that the thickness of such a near-surface layer is comparable with the NP size. If pure toluene is injected into the freshly cleaned cell, no oscillations in the XR profile are observed. Repeating the NP solution injection after re-cleaning and re-assembling the cell reproduces the position and period of the XR profile oscillations. It is therefore clear that contamination effects can be excluded and that the oscillatory character of the XR profile indicates NP accumulation at the solution surface. In order to quantify the above observations, the XR profile is fitted using a physically motivated model for the surface-normal electron density profile. Contrary to the box model described in the previous section, in this physical model approach the electron density profile of the system 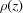 is modelled according to the physical properties of the NPs used and to their arrangement in a monolayer structure. At the basis of this model (Calzolari *et al.*, 2012 ▶) is the fact that the electron density of a monolayer of hexagonally packed identical spheres of radius 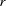, immersion 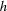 below the toluene surface, and with lateral separation 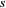 between the surfaces of neighbouring spheres (Fig. 6*b* ▶), is given by
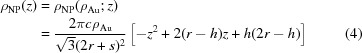
for 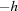 < 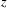 < 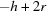, and 0 elsewhere. Here 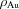 = 4.64 e^−^ Å^−3^ is the electron density of bulk gold, and 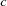 is the coverage parameter describing the fraction of surface occupied by the NP monolayer. The decane-thiol shell surrounding the Au cores is modelled by combining a hollow sphere of density 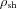 = 0.29 e^−^ Å^−3^ and thickness 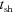, which is concentric with the full sphere of density 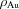 describing the gold cores (Fig. 6*b* ▶). The solvent–vapour interface electron density is modelled with the usual error function,
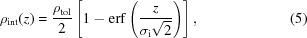
where 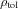 = 0.283 e^−^ Å^−3^ is the electron density of toluene, and 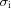 is the toluene–air interface roughness. Gaussian distributions 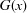 = 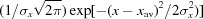 are introduced for the particle radius 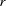 and vertical NP position 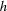, in order to take into account NP size polydispersity and NP vertical disorder, respectively. Thus, the complete physical 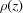 model expression is given by
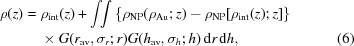
where the subtracted term 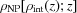 accounts for the liquid displaced by the NPs that accumulate at the toluene surface.

The best fit (blue line in Fig. 6 ▶) obtained by applying the Parratt recursive method (Parratt, 1954 ▶) yields the electron density profile reported in the inset [Fig. 6(*a*) ▶, blue line]. The fit parameters are listed in Table 3 ▶. The fit is obtained by fixing the toluene, gold and decane-thiol electron densities (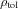, 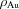 and 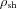, respectively) to their respective nominal values, and the total coverage 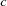 to 1. We let the particle radius 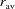, its standard deviation 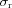 and the thiol shell thickness 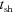 vary in a small range around their nominal values. The toluene–air interface roughness 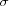 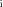 is fitted within a small range centred at the theoretical capillary wave roughness 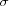 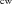. For bulk toluene with our experimental 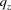 resolution, 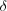 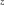 = 0.015 Å^−1^ and, considering the toluene surface tension at 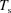 = 300 K (Kahl *et al.*, 2003 ▶), 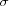 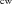 is calculated (Braslau *et al.*, 1988 ▶) to be ∼3.1 Å. The parameter 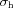 is constrained within the range 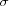 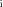 < 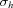 < 2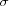 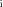, since the NP vertical disorder is correlated with the capillary-wave roughness of the liquid surface. Only the particle average immersion 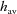 and the neighbouring NP separation 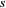 are left completely free during the 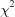 minimization (free fit parameters are emphasized in bold in Table 3 ▶).

The main result of the model fitting is that the XR data are indeed compatible with a single layer of NPs accumulating near the surface of the NP solution. The fitted particle immersion (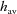 ≃ 5.5 nm) indicates that the particles are mostly immersed in the liquid toluene phase, which is expected due to the fact that toluene is a very good solvent for the NPs. The large average in-plane particle separation obtained from the XR fit (∼14 nm, 5 h after injection) indicates that, within the coherence length probed by the experimental set-up, either the NP monolayer is rather dilute or it is composed of patches of close-packed NPs separated by empty areas. XR is not sensitive to the actual in-plane arrangement of the NPs; however, grazing-incidence small-angle X-ray scattering (GISAXS) data (not shown) do not exhibit the well defined peak that would appear if the particles were close packed, thus supporting a scenario involving a dilute and disordered NP monolayer covering the entire surface of the NP solution.

The second XR profile in Fig. 6 ▶ (squares) was measured three hours after the first one (up-triangles), *i.e.* eight hours after injecting the NP solution into the chamber. The XR oscillation is still noticeable but the oscillation amplitude is less pronounced. The corresponding fit (green lines in Fig. 6 ▶) indicates a further reduction of the NP monolayer density caused by an increase of the average NP separation up to 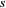 ≃ 20 nm (Table 3 ▶). About 18 h after the injection (diamonds, Fig. 6 ▶) the XR profile no longer exhibits the initial oscillatory features connected to NP surface accumulation: the magenta line is a fit obtained by simply considering a free toluene–air interface with roughness 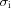 = 5.0 Å. This sequence of XR profiles suggests that the particles, initially accumulated at the solution surface, completely re-dissolve in the bulk solvent phase after a sufficiently long time. The slightly larger toluene–air roughness obtained in our fits (Table 3 ▶) with respect to the expected 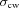 ≃ 3.1 Å is probably linked to the presence of NPs in the vicinity of the solution surface. We also performed high-energy (69.8 keV) XR measurements (down-triangles, Fig. 6 ▶) at the buried NP-solution–sapphire interface, at regular intervals of time, without finding any evidence of NP accumulation or layering near the surface of the solid substrate.

The general scenario that we can infer from these measurements is that, after injection, a part of the particles present in the solution are driven towards the solution–vapour interface, forming a dilute and disordered monolayer. This NP layering effect was reproduced at each new injection of fresh NP solution. Furthermore, the surface NP accumulation is transient, and the NPs slowly re-dissolve into the bulk toluene phase over periods of several hours. We report this totally unexpected finding, although the physical force responsible for the formation of this surface NP layer is not clearly identified, since under normal conditions the particles are expected to be well dissolved in toluene. Shear effects during the injection process cannot account for the particle interfacial accumulation, since the NP dispersion is very dilute (volume fraction ∼10^−7^) and the injection shear rate is only ∼10^−2^ s^−1^. Weak residual charging of the NPs may play a role; however, further investigations are needed to better understand the origin of this phenomenon.

## 5. Fast bulk solvent evaporation for off-equilibrium NP self-assembly   

In this section we describe an example of fast macroscopic solvent evaporation aimed at reaching far-from-equilibrium conditions for NP self-assembly. Several studies showed that far-from-equilibrium conditions can trigger an interfacial NP self-assembly mechanism developing at the receding liquid–vapour interface and yielding extended and highly ordered NP superlattices (Narayanan *et al.*, 2004 ▶; Bigioni *et al.*, 2006 ▶; Bodnarchuk *et al.*, 2010 ▶).

During solvent evaporation, the particles are free to diffuse by Brownian motion in the bulk liquid while it progressively evaporates. The Peclet number, Pe, is the parameter used to gauge the dominance of the Brownian velocity relative to the speed of vertical [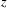 direction in Fig. 1(*a*) ▶] recession of the solution–vapour interface towards the substrate (Routh & Russel, 1999 ▶). Pe is defined as 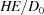, where 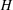 is the initial thickness of the liquid into which the NPs are dissolved, 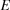 is the velocity of the receding solution surface and 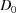 is the Stokes–Einstein diffusion coefficient. For an isolated particle in a homogeneous medium, 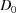 is expressed as 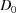 = 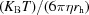, where 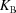 is the Boltzmann constant, 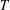 is the temperature, 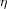 is the medium viscosity and 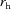 is the hydrodynamic radius of the particle. In our case 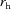 ≃ 45 Å, as obtained by adding the NP core radius to one thiol chain length. For Pe 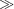 1, the evaporation is dominant and the particles are predicted to accumulate at the solution–vapour interface during evaporation, whereas, for Pe 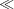 1, Brownian diffusion dominates and the particles are expected to remain uniformly distributed in the bulk solvent (Routh & Russel, 1999 ▶; Gorce *et al.*, 2002 ▶).

**Figure 7:**
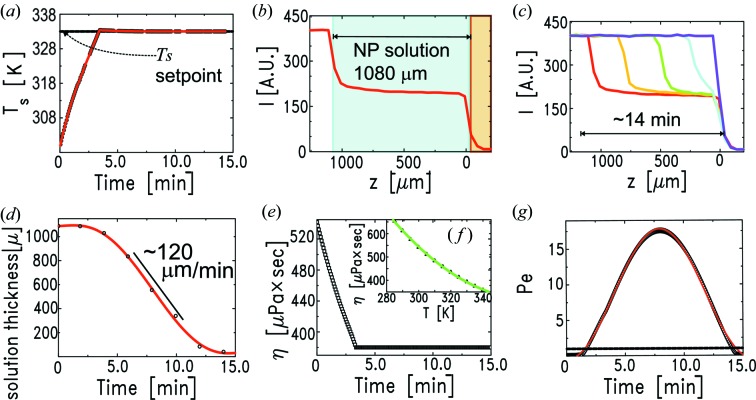
Characterization of the chamber performance in fast bulk solvent evaporation mode. (*a*) The time evolution of the substrate temperature for a 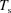 setpoint of 333 K. (*b*) Transmitted X-ray intensity during a vertical (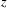) scan of the cell before starting the evaporation. The NP-solution–vapour interface (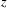 = 1080 µm) and the buried substrate–NP-solution interface (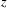 = 0 µm) are highlighted. (*c*) Some of the vertical scans performed during the evaporation of the solvent. Complete macroscopic evaporation takes about 14 min. (*d*) Evolution of the NP-solution–vapour interface vertical 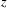 position as a function of time [referring to (*a*)], obtained from the vertical scans presented in inset (*c*). The red line is a sixth-order polynomial fit. (*e*) Time evolution of toluene viscosity 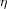 as a consequence of the changing sample temperature [inset (*a*)], neglecting the contribution due to solution volume shrinking. (*f*) Reference values for the toluene 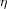 as a function of temperature. (*g*) Evolution of the Pe number during the evaporation process.

With the aim of achieving the highest possible Pe number, we heated the sample using an additional power supply driven by one of the temperature controllers. In this way we could reach the temperature ramp plotted in Fig. 7(*a*) ▶ (red line). The temperature setpoint was fixed at 333 K. We monitored the evaporation process by scanning the cell along the 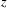 direction through the X-ray beam, and measuring its attenuation profile at regular intervals of time, in order to detect the position of the progressively receding solution–vapour interface (Fig. 7*c* ▶). The initial thickness, before starting the evaporation, was 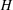 ≃ 1080 µm (Fig. 7*b* ▶). From the vertical scans performed during the evaporation, we extract the NP solution surface position, which is plotted *versus* time in Fig. 7(*d*) ▶. The complete macroscopic evaporation of the solvent took about 14 min, corresponding to an average liquid surface recession speed of ∼95 µm min^−1^, with a maximum value of ∼120 µm min^−1^, reached during the second half of the process.

For the calculation of the Pe number attained during this evaporation process, we neglect the fact that 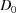 will necessarily decrease as the NP suspension becomes more concentrated upon drying (dilute limit conditions; Gorce *et al.*, 2002 ▶). Instead, we take into account the variation of solvent viscosity 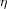 with sample temperature 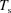. Fig. 7(*f*) ▶ displays the value of 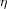 for toluene (symbols) as a function of temperature (Santos *et al.*, 2006 ▶). By interpolation (green line in Fig. 7*f* ▶), and using the available substrate temperatures (Fig. 7*a* ▶), we obtain the evolution of 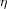 during the evaporation process (Fig. 7*e* ▶). We take into account also the temporal evolution of 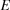, calculated as the derivative of the polynomial fit (red curve) that describes the experimental points (circles) in Fig. 7(*d*) ▶.

The Pe number resulting from this procedure is plotted in Fig. 7(*g*) ▶ (diamonds). The maximum value is ∼18. The black dashed line indicates unity, and in our case Pe is higher than 5 for most of the evaporation process. These values are high enough to warrant the self-assembly to develop at the receding solution–vapour interface (Gorce *et al.*, 2002 ▶). The calculated Pe can be interpolated reasonably well considering a sinusoidal function 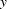 = 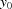 + 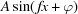, with 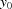 = 8.73, 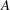 = 9.09, 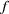 = 0.0067 and 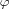 = 1.63 (red line, Fig. 7*g* ▶).

**Figure 8:**
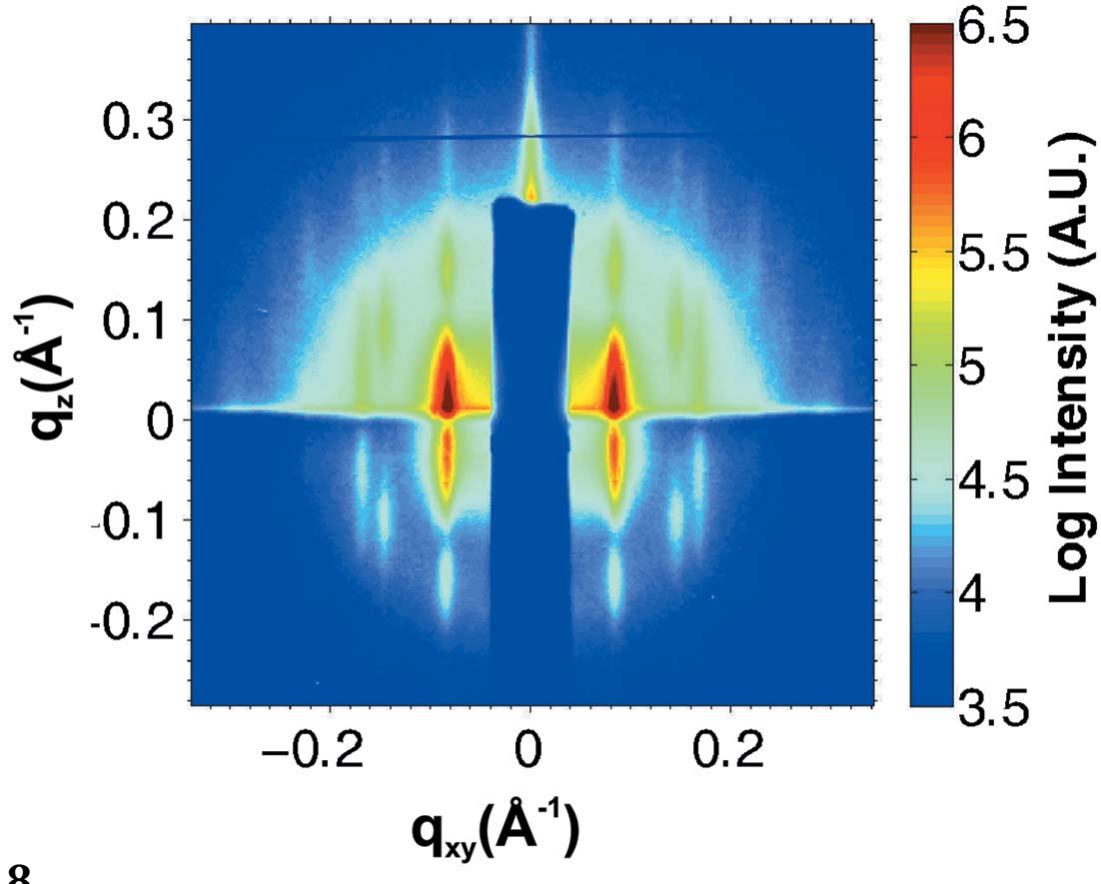
*In situ* GISAXS characterization of a NP superlattice grown by macroscopic fast solvent evaporation and subsequent structural annealing *via* controlled nanoscale solvent adsorption. The initial NP solution contained a mixture of Au NPs of 6.5 nm and 2.8 nm core size in number ratio small/large = 60/40. The organization of the large NPs is predominant, as shown by correlation rods at 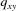 = 0.083 Å^−1^ and clearly discernible higher-order diffraction peaks.

As a demonstrative example, we show the *in situ* GISAXS characterization of a NP superlattice obtained by fast evaporation of a toluene solution containing bimodal NPs comprising 6.5 nm and 2.8 nm Au cores capped with decane-thiols, with size polydispersity of 10% and 25%, respectively, and with number ratio small/large = 60/40. Performing macroscopic fast evaporation at 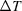 = 35 K followed by a controlled nanoscale solvent adsorption at 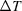 = 1 K leads to the diffraction pattern shown in Fig. 8 ▶. Well defined diffraction peaks reveal a predominant in-plane organization of the large NPs, while ordering signatures for the small NPs are absent. The coordinate of the first-order correlation rods (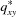 = 0.083 Å^−1^) and the higher-order peaks indicate a well developed in-plane hexagonal arrangement, with lattice constant 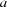 = 2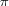/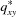 ≃ 76 Å. From the rod width 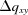 = 0.0113 Å^−1^, it is possible to estimate the in-plane correlation length to 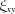 = 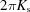/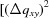 − 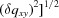 = 660 Å, where 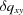 = 0.0036 Å^−1^ is the experimental horizontal resolution and 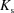 = 1.123 is the Scherrer constant for spherical crystallites (Smilgies, 2009 ▶). We conclude that the in-plane ordered domains present in this NP film have a typical extension of about 8 to 9 lattice constants. More details about this system will be published elsewhere.

## 6. Conclusions   

We have designed and realised an experimental chamber for the study of solvent-mediated NP self-assembly. The chamber adopts a novel approach: the combination of tunable-rate macroscopic solvent evaporation and precisely controlled solvent adsorption/desorption at the nanoscale. The process of NP self-assembly can be characterized throughout all of its phases by *in situ* X-ray scattering techniques (*e.g.* XR and GISAXS), from the initial bulk NP dispersion to the resulting dry film after macroscopic solvent evaporation and/or solvent annealing. In principle, this experimental chamber can be used for static and dynamic X-ray investigations of self-assembly processes involving both NPs and a wide range of additional classes of mesoscopic self-assembling building blocks.

## Figures and Tables

**Table 1:** Thermophysical properties of the materials used to fabricate the sample holder Values for 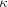 and 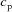 refer to 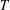 = 298 K, while the reported values for 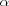 are valid in the range 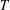 = 273–298 K (Lide, 2004 ▶; Blumm *et al.*, 2010 ▶).

	Thermal conductivity κ (W m^−1^ K^−1^)	Specific heat capacity *c*_p_ (J kg^−1^ K^−1^)	Coefficient of linear expansion α (×10^−6^ K^−1^)
Copper	401	385	16.5
Stainless steel	17	533	17.3
PTFE	0.26	1100	100

**Table 2:** Fitted thicknesses (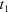, 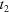) and roughnesses (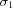, 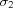) for the two-box model fits of the XR profiles pertaining to pure toluene absorption on flat silicon substrates (Fig. 5 ▶)

Δ*T* (K) (± 1 × 10^−3^ K)	*t*_1_ (Å) (± 0.6 Å)	σ_1_ (Å) (± 0.6 Å)	*t*_2_ (Å) (± 0.6 Å)	σ_2_ (Å) (± 0.6 Å)
11.452	5.1	2.7	5.8	3.0
3.150	5.1	2.7	9.6	3.6
0.963	5.1	2.6	11.3	3.0
0.551	5.1	2.7	13.7	3.2
0.142	5.1	2.7	24.1	3.9
0.060	5.1	2.7	37.4	3.9

**Table 3:** Fitted physical model parameters describing the surface of a NP toluene solution 5 h after injection (Fig. 6 ▶, up-triangles) and 8 h after injection (Fig. 6 ▶, squares) The error associated with the parameters listed in this table is ±1 Å. The parameters left completely free during the 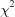 minimization are emphasized in bold.

Physical model parameter	5 h after spreading	8 h after spreading
*r*_av_ (Å)	33	33
σ_r_ (Å)	3	3
*s* (Å)	**137**	**204**
*h*_av_ (Å)	**53**	**54**
σ_h_ (Å)	6	4
σ_i_ (Å)	4	4
*t*_sh_ (Å)	12	12
